# Dsg3 epitope-specific signalling in pemphigus

**DOI:** 10.3389/fimmu.2023.1163066

**Published:** 2023-04-18

**Authors:** Thomas Schmitt, Christoph Hudemann, Sina Moztarzadeh, Michael Hertl, Ritva Tikkanen, Jens Waschke

**Affiliations:** ^1^ Instiute of Anatomy, Faculty of Medicine, Chair of Vegetative Anatomy, Ludwig-Maximilian -Universität (LMU) Munich, München, Germany; ^2^ Department of Dermatology and Allergology, Philipps-University Marburg, Marburg, Germany; ^3^ Institute of Biochemistry, Medical Faculty, Justus-Liebig-University Giessen, Giessen, Germany

**Keywords:** pemphigus, autoimmune disease, skin, epidermis, desmosomes, adhesion, keratin, desmoglein (dsg)

## Abstract

**Introduction:**

Pemphigus is an autoantibody driven disease that impairs the barrier function of the skin and mucosa by disrupting desmosomes and thereby impeding cellular cohesion. It is known that the different clinical phenotypes of pemphigus vulgaris (PV) and pemphigus foliaceus (PF) are dependent on the autoantibody profile and target antigens that, amongst others, are primarily desmoglein (Dsg)1 and/or Dsg3 for PV and Dsg1 for PF. However, it was reported that autoantibodiesagainst different epitopes of Dsg1 and Dsg3 can be pathogenic or not. The underlying mechanisms are very complex and involve both direct inhibition of Dsg interactions and downstream signalling. The aim of this study was to find out whether there is target-epitope-specific Dsg3 signalling by comparing the effects of the two pathogenic murine IgGs, 2G4 and AK23.

**Methods:**

Dispase-based dissociation assay, Western Blot analysis, Stimulated emission depletion microscopy, Fura-based Ca2+ flux measurements, Rho/Rac G-Protein-linked immunosorbent assay, Enzyme-linked immunosorbent assay.

**Results:**

The IgGs are directed against the EC5 and EC1 domain of Dsg3, respectively. The data show that 2G4 was less effective in causing loss of cell adhesion, compared to AK23. STED imaging revealed that both autoantibodies had similar effects on keratin retraction and reduction of desmosome number whereas only AK23 induced Dsg3 depletion. Moreover, both antibodies induced phosphorylation of p38MAPK and Akt whereas Src was phosphorylated upon treatment with AK23 only. Interestingly, Src and Akt activation were p38MAPK-dependent. All pathogenic effects were rescued by p38MAPK inhibition and AK23-mediated effects were also ameliorated by Src inhibition.

**Discussion:**

The results give first insights into pemphigus autoantibody-induced Dsg3 epitope-specific signalling which is involved in pathogenic events such as Dsg3 depletion.

## Introduction

The skin and mucosa provide an effective barrier against external hazards such as pathogens or harmful chemicals. Pemphigus is an autoantibody driven disease that impairs barrier integrity by disturbing the turnover of desmosomes. Desmosomes provide mechanical cohesion between neighbouring epithelial cells (1). As a result, liquid-filled blisters are formed within the epithelia, which can affect a large portion of the skin or mucous membranes. The blisters can thus cause severe complications and high mortality, mostly due to barrier defects (2). Desmosomes consist of the cadherin adhesion molecules desmoglein (Dsg) and desmocollin (Dsc) subfamily. These are anchored to keratin filaments by adaptor proteins such as plakoglobin (Pg) and plakophilin (Pkp) and the linker protein desmoplakin (Dp). It is well known that the clinical pemphigus phenotypes correlate with the autoantibody profile. The most common pemphigus variants are pemphigus vulgaris (PV) characterized by autoantibodies against Dsg3, affecting the mucosa only or against Dsg1 and Dsg3, affecting the mucosa and epidermis and pemphigus foliaceus (PF) characterized by autoantibodies against Dsg1, affecting the epidermis exclusively (3–5). However, the pathomechanisms causing the loss of adhesion are quite complex (6). For anti-Dsg3 antibodies, it was shown that both direct inhibition of Dsg interaction (7–9) and signalling events induced by the autoantibody binding play a major role (10, 11). In this context, it was reported that several kinases and other signalling proteins relevant for pemphigus pathogenesis form complexes with desmosomal proteins (12, 13). Recently, interaction of Erk1 and p38MAPK with Dsg1 was confirmed by BIO-ID (14).

For anti-Dsg1 autoantibodies, direct inhibition was not found in all studies (7, 15–17). Frequently, autoantibodies against other antigens are also found (18). Autoantibodies against Dsc1, Dsc2 or Dsc3 were also reported to be pathogenic (19–26). However, the role of autoantibodies against other targets is less clear. It is hypothesised that they cause additive effects, thereby potentiating the pathogenic effects of the autoantibodies against the desmosomal cadherins (27–29).

It was reported that different anti-Dsg antibodies do not always show a similar degree of pathogenicity (7, 15–17). Dsg isoforms consist of five extracellular domains (EC1-EC5), similar to other cadherins. These share a high structural homology, except for the membrane proximal EC5 domain (30). Several studies reported that most pemphigus autoantibodies bind to the N-terminal EC1-domain, the major mediator of homo- and heterophilic interactions (31, 32). Approximately 50 % of PV patients exhibit detectable serum IgGs recognizing the EC1 domain, followed by EC2 (26 %), EC4 (21 %), EC5 (17%) and EC3 (15%) (33). Antibodies directed against EC1 or EC2 are known to exhibit pathogenic effects, whereas those targeting EC5 are reported to be mostly non-pathogenic (34, 35). It was reported that healthy individuals from endemic groups with an increased risk of PF onset often showed increased levels of non-pathogenic anti-Dsg1-IgGs (35–38). These were exclusively IgG2 whereas pathogenic autoantibodies were usually of IgG4 subtype (39). However, non-pathogenic and pathogenic antibodies can often share the same light or heavy chains (34). It is even hypothesised that pathogenic pemphigus autoantibodies can evolve from non-pathogenic antibodies via epitope spreading (34, 35, 40). Epitope spreading from Dsg3 to Dsg1 is described in the relatively frequent transformation from mucosal-dominant (anti-Dsg3-IgG only) to mucocutaneous PV (anti-Dsg1- and anti-Dsg3-IgG). The same is true for a less frequent transformation from PF (anti-Dsg1-IgG only) to PV (anti-Dsg1- and anti-Dsg3-IgG) (41, 42). A further piece of evidence is the observation that the introduction of a single Dsg3-reactive T-cell was sufficient to induce the expression of polyclonal anti-Dsg3 IgG in mice *in vivo* (43). Pemphigus patient sera usually display a complex polyclonal mixture of autoantibodies that in most, but not all cases show a good correlation with the disease severity (44, 45).

**Figure 1:**
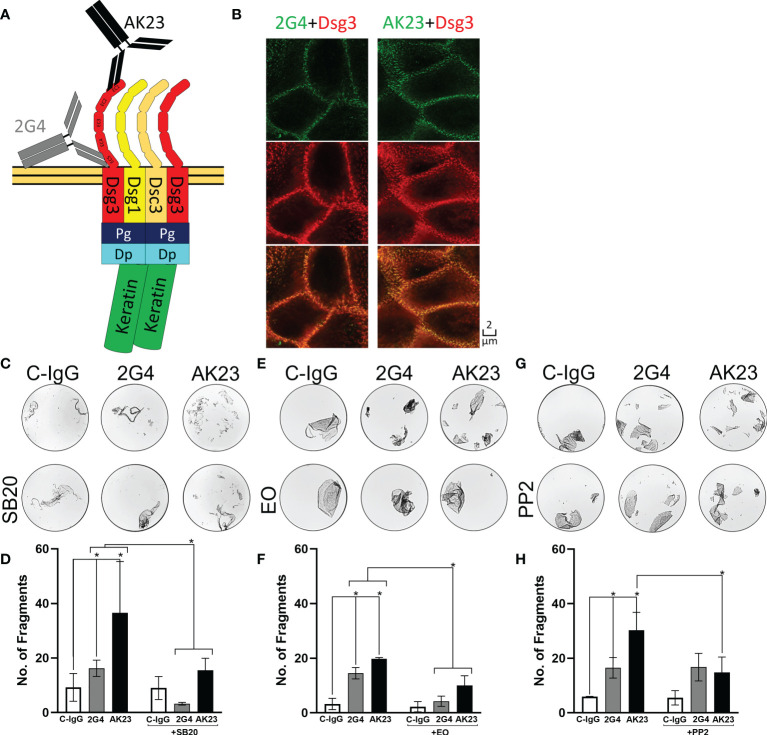
2G4 is a pathogenic Dsg3 specific IgG. **(A)** Schematic depiction of binding positions of AK23 (Black), directed against the EC1 domain and 2G4 (Grey) directed against the EC5 domain of Dsg3, on a desmosome-bound Dsg3. **(B)** Co-staining of either 2G4 or AK23 (green) with Dsg3 (intracellular domain, red) and an overlay of both channels in HaCaT cells after 24 hours incubation with the IgGs (1:50), imaged with the STED microscope (N=4). **(C)** Representative images of dispase-based dissociation assay results in HaCaT cells. Vehicle control (left) pre-treated with SB20 (60 µM, inhibiting p38MAPK, right). **(D)** Quantification of dispase-based dissociation assay results using SB20 (N=4). **(E)** Representative images of dispase-based dissociation assay results in HaCaT cells. Vehicle control (left) pre-treated with EO (40 nM, inhibiting p38MAPK, right). **(F)** Quantification of dispase-based dissociation assay results using EO (N=4). **(G)** Representative images of dispase-based dissociation assay results in HaCaT cells. Vehicle control (left) pre-treated with PP2 (10 µM, inhibiting Src, right). **(H)** Quantification of dispase-based dissociation assay results using PP2 (N=4). * indicates statistically significant differences in two-way-ANOVA with Sidaks correction for multiple comparisons p<0.05.

Due to their high prevalence in pemphigus patients and their high pathogenicity in several *in vitro* and *in vivo* studies, EC1-specific antibodies such as the mouse monoclonal IgG1 AK23 are typically used to investigate PV pathology (**Figure 1A**) (46). Recently, a pathogenic mouse monoclonal anti-Dsg3 IgG called 2G4, directed against the EC5 domain, was developed (47). The aim of this study was thus to directly compare the two antibodies to clarify if autoantibodies in PV can induce a Dsg3 epitope-specific signalling response.

## Results

### AK23 is more effective than 2G4 in reducing cell-cell adhesion

Using immunostaining and super resolution stimulated emission depletion (STED) microscopy showed that both 2G4 and AK23 produced a Dsg3 staining pattern comparable to a commercial polyclonal IgG directed against the intracellular domain of Dsg3 (**Figure 1B**). In the dispase-based dissociation assay, 2G4 induced a significant loss of adhesion, compared to the control using IgG purified from the serum of healthy volunteers (C-IgG). However, 2G4 showed a 2.54-fold weaker effect on the adhesion of HaCaT cells than AK23 (1.98-fold without subtracting background fragments under control conditions). The effects of both IgGs were ameliorated by inhibition of p38MAPK, a kinase central for pemphigus pathogenesis (6), using SB202190 (SB20) (**Figures 1C, D**) or EO1428 (EO) (**Figures 1E, F**). Inhibition of Src, using PP2, on the other hand was effective in reducing AK23-induced effects, but had no effect on fragment numbers under 2G4 treatment (**Figures 1G, H**).

**Figure 2:**
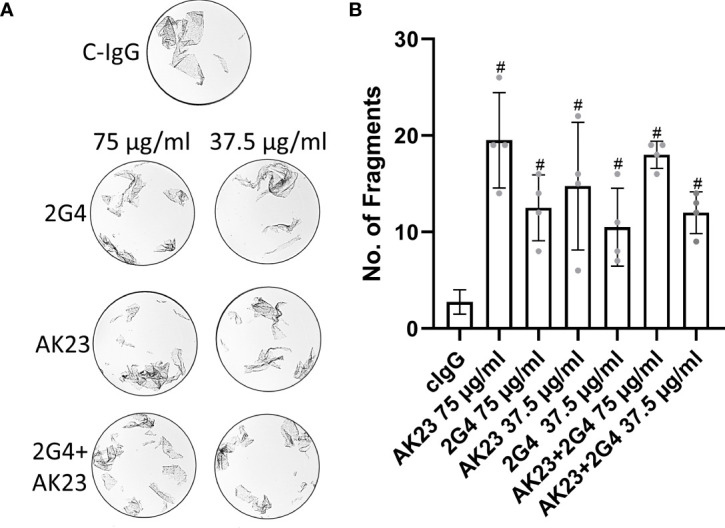
**(A)** Representative images of dispase-based dissociation assay results in HaCaT cells. Control, treated with C-IgG only (top), treated with either single 2G4, AK23 or a mixture of both at a concentration of 75 µg/ml eacht (left) and at half concentration of 37.5 µg/ml right. **(B)** Quantification of dispase-based dissociation assay (N=4). # indicates statistically significant differences towards control conditions, one-way-ANOVA with Dunett correction for multiple comparisons, p<0.05.

To check if there are additive and polyclonal effects potentially increasing the loss of adhesion, we used mixtures of AK23 and 2G4 in dispase dissociation assays. A mixture of AK23 and 2G4 at a concentration of 75 µg/ml each did not lead to a significant increase in the number of fragments, compared to AK23 alone at a concentration of 75 µg/ml (as applied in all other experiments). A mixture of AK23 and 2G4 at a concentration of 37.5 µg/ml for both led to a number of fragments similar to the number of fragments induced by 2G4 alone at a concentration of 75 µg/ml (**Figures 2A, B**).

### Effects of 2G4 and AK23 on the organization of keratin filaments and distribution of Dsg3 and Dp

**Figure 3:**
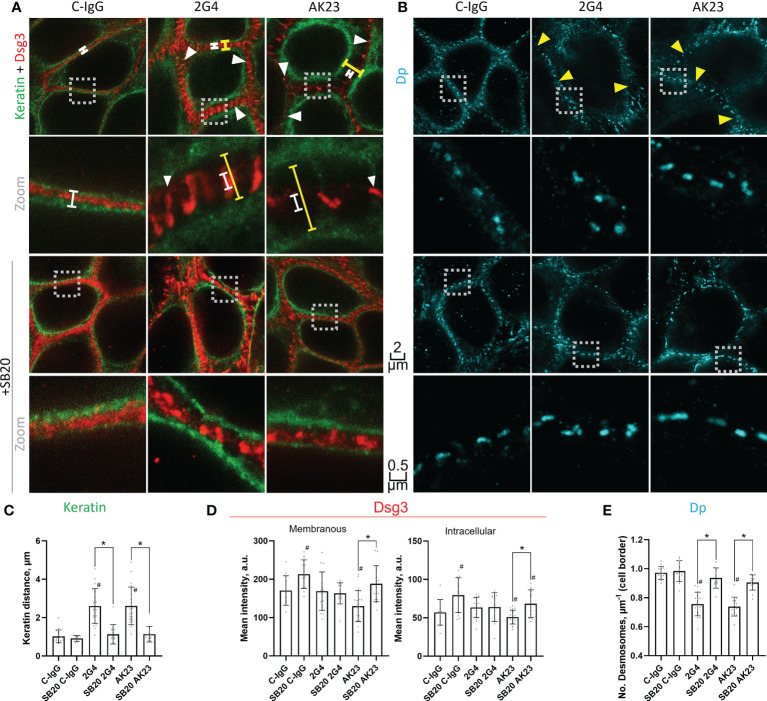
STED microscopy images reveal specific pathogenic effects of 2G4 and AK23. **(A)** Co-staining of Dsg3 (red) and pan-cytokeratin (green) in HaCaT cells treated with either C-IgG, AK23 or 2G4 for 24 h after 1 h preincubation with vehicle or SB20 (60 µM, inhibiting p38MAPK). Spans illustrate the degree of keratin filament retraction (yellow) compared to control (white). White arrowheads showcase the fragmented staining of Dsg3, which is mostly associated with keratin filaments. **(B)** Staining of Dp (with anti-Dp-pAb, A7169) after 24 h treatment with IgGs and 1 h preincubation with vehicle or SB20. Yellow arrowheads indicate reduced numbers of desmosomes per µm of cell border. Two different magnifications are shown for each condition (Scalebars as indicated, boxes with dotted outline indicate the area which was zoomed in on); Quantification of the STED images: **(C)** Keratin filament retraction form the cell borders towards the nucleus. **(D)** Dsg3 staining intensity at the cell borders/membranes (left) or in the cytoplasm (right). **(E)** Number of desmosomes per µm membrane (Experiments (N)=4-6, Evaluated images (n)=12-22). * marks statistically significant differences between the two conditions indicated, # indicates statistically significant differences towards control conditions, both in two-way-ANOVA with Sidaks correction for multiple comparisons, p<0.05.

Using STED microscopy, we observed that both 2G4 and AK23 caused fragmentation of Dsg3 staining along cell borders (**Figure 3A**, **S1A**). In addition, following incubation with AK23 the staining intensity of Dsg3 was significantly reduced, both along the cell borders and in the cytoplasm. This effect was not observed for 2G4, showing that AK23 led to a stronger redistribution and loss of Dsg3 than 2G4. The effects were ameliorated by inhibition of p38MAPK using either SB202190 (**Figures 3A, D**) or EO1428 (**Figures S1A, D**). p38MAPK inhibition with SB202190 under control conditions significantly increased Dsg3 staining intensity both at the cell border and in the cytoplasm whereas effects of EO1428 were not significant. This is indicating that p38MAPK is involved in Dsg3 turn-over under basal conditions as well (**Figures 3A, D**; **S1A, D**). Furthermore, a significant retraction of keratin filaments from the cell borders towards the nucleus, another typical pemphigus hallmark, was observed after incubation with either 2G4 or AK23. The effect was similar in extent for both IgGs and was also blocked by inhibition of p38MAPK with either SB202190 (**Figures 3A, C**) or EO1428 (**Figures S1A, C**).

In addition to Dsg3 and keratin filaments, the desmosomal plaque protein Dp was stained and investigated using STED microscopy. Neither of the two IgGs caused significant changes in desmosome morphology (**Figures 3B**; **S1B**). Due to the regular size and shape of desmosomal plaques, this staining was also utilized to determine the number of desmosomes per µm of cell border. Both 2G4 and AK23 caused a similar reduction of desmosome number, comparable to results for PV-patient skin (12). This effect was also abrogated by inhibition of p38MAPK with either SB202190 (**Figure 3B, E**) or EO1428 (**Figure S1B, E**).

### 2G4 and AK23 induce overlapping, yet distinct signalling responses

**Figure 4:**
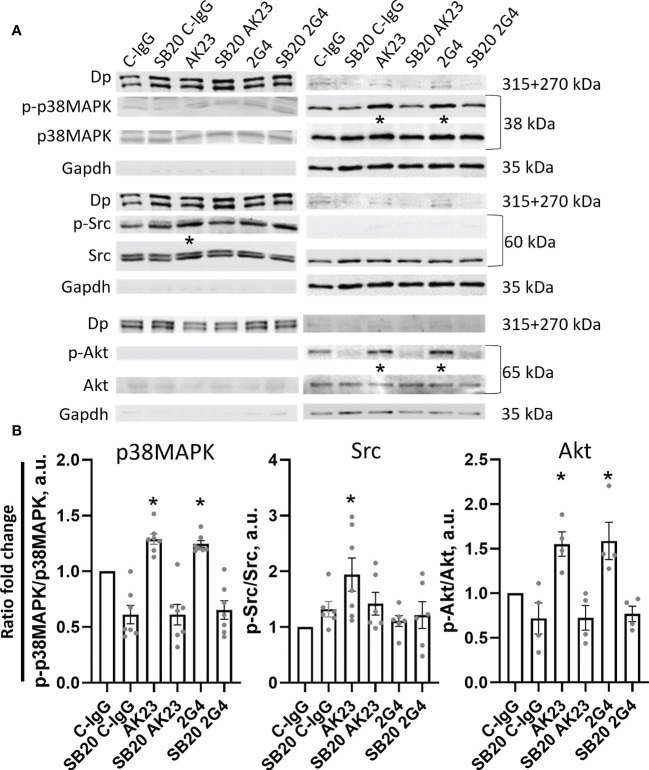
Western blot analysis reveals anti-Dsg3 epitope-specific kinase phosphorylation. **(A)** Representative Western blot images, with respective controls for Triton-soluble (membranous+cytosolic fraction, right) Gapdh and Triton-insoluble (cytoskeleton/desmosome-bound fraction, left) Dp. The cells were treated with either C-IgG, AK23 or 2G4 with or without pre-treatment with SB20 for 1 h, to inhibit p38 MAPK. **(B)** Quantification of differences in phosphorylation of the investigated proteins from the WB results. Significant changes were observed and quantified in the soluble fraction for p38MAPK and Akt and the insoluble fraction for Src (N=4-7). * marks statistically significant differences between the two conditions indicated in two-way-ANOVA with Sidaks correction for multiple comparisons, p<0.05.

Next, we analysed the signalling pathways induced by 2G4 and AK23 respectively, using Western blot analyses after Triton X protein fractionation. Similar to previous studies, p38MAPK was significantly phosphorylated in the Triton X soluble (not cytoskeleton-bound fraction) and both IgGs induced a similar degree of p38MAPK phosphorylation. This effect was ameliorated upon treatment with the p38MAPK inhibitor SB202190 (**Figures 4A, B**) or EO (**Figures S2A, N**). Interestingly, Src, which is another well-known Dsg3-dependent signalling protein involved in loss of cell adhesion in pemphigus (48, 49), was significantly phosphorylated upon treatment with AK23 but not following 2G4 incubation. The effect was dependent on p38MAPK since treatment with SB202190 (**Figures 4A, B**) or EO 1428(**Figures S2A, B**) abolished this effect. As a third target of interest, Akt as downstream kinase of PI3-kinase, which is regulated by the Dsg3-dependent EGFR signalling pathway (6, 11, 50), was investigated. Akt phosphorylation was significantly increased to a similar degree after treatment with either 2G4 or AK23. Inhibition of p38MAPK with SB202190 (**Figures 4A, B**) or EO1428 (**Figures S2A, B**) abolished this effect. Neither of the two antibodies showed a significant impact on the activity of RhoA or Rac1 GTPases (**Figure S3**).

**Figure 5:**
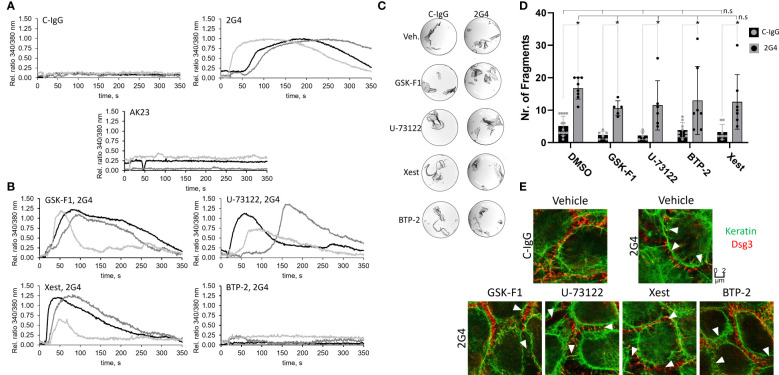
Alterations of intracellular Ca^2+^ signalling induced by 2G4. **(A)** Changes in intracellular Ca^2+^-concentration determined in HaCaT cells, after addition of 2G4, AK23 or C-IgG, by a measured change in ratio of 340/380 nm fluorescence of intracellular FURA-2AM upon Ca^2+^ binding (One curve represents one independent experiment, N=3, obtained from the mean of 8 cells, n=8). **(B)** Changes in intracellular Ca^2+^-concentration after addition of 2G4 to HaCaT cells previously treated with inhibitors of the anti-Dsg1-IgG-dependent Ca-influx pathway (see **Table S2**). **(C)** Representative images of dispase-based dissociation assay results after pre-treatment of HaCaT cells with Ca^2+^-influx pathway mediators for 1 h. **(D)** quantification of the dispase-based dissociation assay results (N=5-13, * makrs statistically significant differences between the two conditions indicated, n.s. (not significant) indicates, that there are no significant differences to the corresponding C-IgG (negative) or 2G4 (positive) control condition, both in two-way-ANOVA with Sidaks correction for multiple comparisons, p<0.05). **(E)** STED images of cells pre-treated with vehicle or Ca^2+^-influx pathway mediators for 1 h and C-IgG or 2G4 for 24 h. White arrowheads indicate fragmented Dsg3 staining.

In addition to protein phosphorylation, the Ca^2+^-influx after treatment with either 2G4 or AK23 was monitored. 2G4 induced a rapid influx of Ca^2+^ into the cells whereas AK23, similar to a previous report, did not (51) (**Figure 5A**). 2G4 did not show any signs of cross reactivity with Dsg1 (**Figure S4**), indicating a signalling mechanism dependent on Dsg3 in this case. This phenomenon was investigated further, employing mediators used in a previous study (51) (**Table S2**). However, in contrast to Dsg1-IgG-dependent signalling (51), inhibition of PI4K (with GSK-F1), PLC (with U-73122) or IP3R (with Xestospongin C) was not effective in preventing the 2G4-mediated Ca^2+^-influx. Rather, inhibition of the downstream calcium release-activated channel (CRAC) (with BTP-2) blocked Ca^2+^-influx, indicating that 2G4 induced a different pathway of Ca^2+^-influx into the endoplasmic reticulum (**Figure 5B**). Next, we tested whether this Ca^2+^-influx is involved in loss of cell adhesion caused by 2G4. However, none of the mediators including BTP-2 prevented any of the pathological changes, including loss of adhesion in the dispase-based dissociation assay (**Figures 5C, D**), keratin filament retraction and fragmentation of Dsg3 staining as revealed by STED microscopy (**Figure 5E**).

## Discussion

### Differences in the pathogenic effects between 2G4 and AK23

We found that 2G4 induced effects typical for pemphigus pathogenesis including loss of adhesion, keratin filament retraction and fragmentation of Dsg3 staining, which is in line with previous data (47). In addition, we observed that 2G4, similar to AK23, induced a decrease in the number of desmosomes per µm of cell border. This is important because loss of desmosomes is a hallmark in pemphigus patient lesions (12, 52). The same was observed by using transmission electron microscopy of human skin treated with PV-IgG *ex vivo.* However, AK23 did not cause significant loss of desmosomes in intact full skin (53). The results of the current study show that 2G4 was more than two times less effective than AK23 in inducing loss of cell adhesion. Despite this, STED imaging revealed a similar degree of keratin filament retraction and loss of desmosomes for the two IgGs. The difference may still be explained in part by the loss of Dsg3 staining caused by AK23, but not 2G4, both along the cell membrane and within the cytosol, indicating Dsg3 degradation. In fact, a complete lack of extradesmosomal Dsg3 following incubation with AK23 was observed, whereas in 2G4-treated cells, Dsg3 was present in linear streaks between adjacent cells. This is also in agreement with previous findings, indicating that the extradesmosomal pool of Dsg3 is affected first, without major changes in desmosome composition (12, 54–57). It also fits to the finding that the loss of adhesion and the widening of the intercellular distance is actually initiated in the spaces between the desmosomes (58, 59) and the desmosomes are affected only later (12, 60–63). Consistent with these reports, the morphology of the desmosomes did not appear to be altered.

The fact that 2G4 induces the typical hallmarks of pemphigus pathology despite being directed against the EC5 domain is interesting because antibodies against most other domains than EC1 or EC2 and particularly those targeting the EC5 domain of Dsg3 or Dsg1, were reported to be mostly non-pathogenic (32, 34, 35, 64). For anti-Dsg1-IgGs found in individuals from an endemic population with high risk to develop PF, EC5-specific IgGs were reported to cause no symptoms. Individuals carrying anti-EC1 or anti-EC2 IgGs on the other hand suffered from clinically evident PF (39). Moreover, anti-Dsg3-IgGs isolated from two patients also demonstrated no pathogenic effects for any of the IgG fractions recognizing only EC5 (32). Furthermore, it was reported that IgGs against EC4 and EC5 are rarely found in clinically evident cases and only appear in rare atypical pemphigus cases, if at all. No patients were described to exclusively have IgGs against the EC5 domain, while patients with IgGs exclusively or mostly targeting EC1 and/or EC2 are common (33, 65). The lack of EC4 and EC5-specific IgGs in patients may be explained by the lower accessibility of these domains since the regions closer to the membrane are less exposed and are thus less likely to be subject to immunological processes, at least within mature desmosomes.

### Differences in the pathogenic effects between 2G4 and AK23 are potentially caused by epitope-specific Dsg3 signalling

Signalling is known to be a major mechanism involved in pemphigus pathogenesis and was found to significantly contribute to blistering in human epidermis (66, 67). Stabilizing keratinocyte desmosomal adhesion may thus represent a novel additive treatment paradigm for pemphigus (68, 69). We proposed previously that autoantibody-specific signalling may contribute to the different clinical phenotypes of pemphigus (6). In line with this, we observed that 2G4 and AK23 induced p38MAPK activation. Akt as downstream target of EGFR and PI3-kinase was also phosphorylated and can thus also be considered to be activated in a Dsg3-dependent manner.

In addition to that, for the first time, we observed that Dsg3-mediated signalling is in part epitope-specific. Src was phosphorylated only after incubation with AK23 but was unaffected by 2G4. This difference in signalling response might also provide an explanation for the observed differences between the two IgGs in degree of loss of adhesion and their effect on the Dsg3 distribution and dynamics. It is likely that extradesmosomal Dsg complexes are degraded in response to AK23, since Dsg3, E-Cadherin and Pg containing complexes were reported to be regulated in a Src-dependent manner (48, 70, 71). This would impact adhesion via alterations in the actin cytoskeleton which was reported to stabilize extradesmosomal Dsg3 binding events (72).

Interestingly, the phosphorylation of both Src and Akt was dependent on p38MAPK activity. However, previous reports indicated that p38MAPK can also be regulated by Src, indicating a feedback loop (49, 73). Src seems to regulate the actin dynamics (48, 70, 71), p38MAPK on the other hand was reported to regulate keratin dynamics (74–77). However, p38MAPK was mostly found to be located in the soluble (non-desmosome-bound) pool and to be activated by depletion of Pg. Src on the other hand was confined mostly to the cytoskeleton bound (desmosomal) fraction, as also shown previously (78). Since it was proposed that autoantibodies in pemphigus first induce signalling via extradesmosomal Dsg molecules (79), it may be that Src activity is regulated by p38MAPK activity via keratin restructuring and p38MAPK activity at least in part by Src activity via actin restructuring.

These data support the hypothesis that p38MAPK is a central pathway in pemphigus pathogenesis, which in epidermal keratinocytes orchestrates other signalling events involved in skin blistering. The current results together with previous finding may explain, why p38MAPK inhibition alone was effective to abrogate PV-IgG-induced skin blistering *in vivo* and in human skin ex vivo (53, 80). Inhibition of Src alone on the other hand was protective against the effects of AK23 but not PV-IgG in human epidermis (49). This might be a result of the activation of Dsg1-dependent pathways via anti Dsg1-IgG, but it is now clear, that this effect could also be epitope specific and dependent on the presence of the right mixture of polyclonal IgGs. In contrast to that, for the formation of mucosal erosions, p38MAPK appears to not be critical. Inhibition of p38MAPK alone was not sufficient to prevent blister formation in human mucosa *ex vivo* (81). This indicates that the interplay of several different pathways is important for a significant loss of adhesion and blister formation *in vivo*. Both p38MAPK and Src are major players in this context and seem to be interdependent at least to some degree but can possibly also act independent of each other. This dynamic seems to be regulated at least in part in an epitope specific manner.

The second example for epitope-specific signalling we observed was that 2G4 induced a significant degree of rapid Ca^2+^-influx, whereas AK23, similar to previous findings, did not (17, 51). Since 2G4 showed no cross-reactivity with Dsg1, this most likely is a Dsg3-dependent mechanism. Inhibitors of the known Dsg1-dependent Ca^2+^-influx pathway revealed that CRAC was involved in this effect whereas PI4K, PLC and IP3R were not. Because inhibition of CRAC did not show any protective effect on 2G4-mediated loss of cell adhesion, we conclude that Ca^2+^-influx by this mechanism does not play a major role in pemphigus pathogenesis, which is different to the anti-Dsg1-induced Ca^2+^signalling, which reduced cell adhesion *via* PI4K, PLC, IP3R and CRAC (51).

Previous studies reported that the role of p38MAPK for loss of keratinocyte adhesion and epidermal blister formation might be dependent on polyclonal effects of anti-Dsg1- and/or anti-Dsg3-IgGs (82, 83). At least in combination with the bacterial exfoliative toxin A, which specifically cleaves Dsg1, much lower concentrations of AK23 of 10-15 µg/ml (compared to the 75 µg/ml used in the current study) were sufficient to cause loss of adhesion in a p38MAPK- and tyrosine kinase-independent manner. Moreover, a monoclonal PF-IgG was sufficient to cause epidermal blistering in a p38MAPK-independent manner. Therefore, we tested whether additive or polyclonal effects of AK23 and 2G4 on loss of cell adhesion were present when both antibodies were applied in combination. However, effects were similar when either the full or half of the concentration was used for both autoantibodies. These results indicate that polyclonal mixtures of these two antibodies did not induce additive effects on loss of adhesion and that the concentrations used are effective to induce maximal loss keratinocyte adhesion which is blunted by p38MAPK inhibition. Moreover, a single pathogenic autoantibody directed against Dsg3 is sufficient to induce an epitope-specific signalling response, at least at the concentrations used in this study. However, this does not rule out that combinations with other autoantibodies, which alone are considered to be non-pathogenic, may alter the signalling response or the extent of Dsg1/Dsg3 clustering and depletion (55, 82, 83).

## Conclusion

The study supports the notion that monoclonal Dsg3-specific autoantibodies such as 2G4 and AK23 are pathogenic (47, 84) and are effective to cause loss of keratinocyte adhesion and induce reorganization of keratin filaments and loss of desmosomes. However, the pathogenicity is dependent on the target epitope and maybe explained in part by epitope-specific signalling including Src which may be involved in Dsg3 degradation. Further studies are required to determine how epitope-specific signalling is elicited upon autoantibody binding.

## Materials and methods

### Cell culture

The human epidermal keratinocyte cell line HaCaT was used for all experiments. The cells were cultivated in a humidified, 5% CO_2_ atmosphere at 37°C in Dulbecco’s Modified Eagle Medium (DMEM) (Life Technologies, CA) with 10 % FCS (Biochrom, DE) and 50 U/ml penicillin and 50 µg/ml streptomycin (both AppliChem, DE). They were passaged using a trypsin-EDTA solution (Merck, DE). For experiments, the cells were pre-incubated with the mediators (**Table S2**) for 1 h and with the IgGs for 1 h for signalling or 24 h for other experiments. Both AK23 and 2G4 were used in a concentration of 75 µg/ml, IgG purified from healthy volunteers was used 1:50.

### Ratiometric intracellular Ca^2+^ measurements

Fura-2AM (Thermo Fisher, USA) was used to measure intracellular Ca^2+^ in real time. The cells were grown in an 8-well µ-slide (Ibidi, DE). Mediators (**Table S2**) were contained in all incubation steps and during the measurement. A mix of 1 µmol/l Fura-2AM and 0.02 % pluronic (Thermo Fisher, USA) was applied for 20 min in measurement buffer (140 mmol/l NaCl, 3.6 mmol/l KCl, 2.6 mmol/l CaCl_2_(H_2_O)_2_, 0.5 mmol/l MgSO_4_, 0.5 mmol/l NaH_2_PO_4_(H_2_O)_2_, 2 mmol/l NaHCO_3_, 5 mmol/l HEPES and 5 mmol/l D+Glucose, pH 7.35) at 37°C. The cells were washed twice with measurement buffer. Measurements were performed using MetaFluor (Moleculardevices, USA) on an Axio Observer A1 (Zeiss, DE) with a Polychrome V (Till Photonics, DE), a CoolSNAP-Hq2 digital camera (Photometrics, USA) and a Fura-2 filter set. For each independent experiment, the signals from 8 out of 15 randomly selected cells were evaluated (occasional non-responding cells were not included, very rare oscillating cells and weak responders were included, N = 4).

### Cell lysis, gel electrophoresis and Western blotting

Cells were cultured in 24-well-plates. Lysates were fractioned into a soluble and insoluble fraction using Triton-extraction-buffer (0.5% Triton X-100, 50 mmol/l MES, 25 mmol/l EGTA, 5 mmol/l MgCl_2_, pH 6.8, 0.1% pepstatin+aprotinin+leupeptin, 1% Phenylmethylsulfonylfluorid) for 10 min on ice under gentle shaking. The pellet was separated at 14.000 rpm for 10 min at 4°C and the supernatant was retrieved. The pellet was washed once and lysed with ultrasound in SDS lysis buffer (25 mmol/l HEPES, 2 mmol/l EDTA, 25 mmol/l NaF, 1% SDS, pH 7.6, complete™ (Merk, USA)). The protein amount was determined with a commercial Pierce BCA protein assay kit. Western-blotting was performed, using a standard wet blotting protocol on nitrocellulose membranes (Life Technologies, USA). Membranes were blocked with ROTI^®^Block (Carl Roth, DE) 1:10 in Tris-buffered saline with 0.05% tween (TBST) for 1 h. The Antibodies (**Table S1**) were used overnight at 4°C in 5% BSA in TBST anti-rabbit/mouse horseradish-peroxidase-coupled secondary antibodies (Dianova, DE) were used 1:10.000 in TBST for 1 h and visualized with self-made ECL solution on a FluorchemE developer (Protein Simple, USA).

### Dispase-based dissociation assay

After incubation as described above, confluent cell monolayers were washed with Hank´s buffered saline solution (HBSS) and subjected to 2.4 U/ml dispase II (Sigma Aldrich, USA) in HBSS for 20 min at 37 °C, 95% humidity and 5% CO_2_. After detachment of the monolayer the reaction was stopped by adding 200 µl HBSS. Defined shear stress was applied with an electrical pipette. Images for fragment counting were taken using a binocular microscope (Leica, DE) and an EOS 600D camera (Canon, Japan).

### Immunostaining

Samples were washed and fixed with ethanol (-20°C) shaking on ice for 30 min followed by acetone (-20°C) for 3 min. The samples were blocked with 3% bovine serum albumin (BSA) and 1% normal goat serum in PBS for 30 min. Primary antibodies were applied over 3 h at RT STAR-RED- or Alexa 594-coupled goat-anti-rabbit/mouse secondary antibodies (Abberior GmbH, DE) were incubated for 1 h and DAPI 1:10.000 for 15 min. The coverslips were mounted on glass slides using Prolong™ Diamond Antifade Mountant (Thermo Fisher GmbH, DE).

### STED microscopy

The samples were imaged using an Abberior 3D Stimulated emission depletion (STED) confocal microscope with IMMOIL-F30CC (Olympus GmbH, DE) Star Red and Alexa 594 were excited at 638 nm and 594 nm respectively, using pulsed diode lasers (PDL 594, Abberior Instruments; PiL063X, Advanced Laser Diode Systems). Fluorescent molecules were depleted at 775 nm with a pulsed fibre laser (PFL-P-30-775B1R, MPB Communications) and the emission was detected with an avalanche photodiode detector at 605-625 and 650-720 nm range.

### Statistical analysis

Data were analysed using two-way-ANOVA followed by Sidak-post-hoc-test for multiple comparison or one-way-ANOVA followed by Dunnett-post-hoc-test for multiple comparison, using Graphpad Prism (Graphpad Software, USA). Error bars represent SEM. Significance was assumed with p ≤ 0.05. Data are shown as mean ± SEM. Each N represents one independent experiment, each n one technical replicate.

## Data availability statement

The original contributions presented in the study are included in the article/**Supplementary Material**. Further inquiries can be directed to the corresponding author.

## Author contributions

TS: 65%, conceptualizing the project, performing experiments, performing data evaluation writing the manuscript, conceptualizing and designing the Figures, revising the manuscript. CH: 18%, providing materials, performing experiments, performing data evaluation, checking the manuscript, revising the manuscript. SM: 3%, performing experiments, performing data evaluation. MH: 2%, providing materials, checking the manuscript. RT: 2%, providing materials, checking the manuscript. JW: 10%, providing funding, conceptualizing the project, checking the manuscript, revising the manuscript. All authors contributed to the article and approved the submitted version.

## In memoriam of Detlef Zilikens

The authors all knew Detlef Zilikens as an inspiring scientist. Jens Waschke (JW) listened to his lectures in dermatology when he was a student at Würzburg medical school where he found professor Zillikens to be a very student-friendly and relaxed teacher, which was not applying to all professors he met before. During these lectures, JW heard for the first time about pemphigus. He felt that for this disease the pathogenesis seemed rather simple. It was known that autoantibodies targeting adhesion molecules in desmosomes, the latter he knew well from his anatomy classes, were pathogenic. During his internship in internal medicine in 2001, JW again met Detlef Zillikens to initiate a research project together with Enno Schmidt on the pathogenic effects of autoantibodies in pemphigus. Soon, everything turned out not to be that simple and the pathogenesis of pemphigus not completely clarified. Since the background in the anatomy group on pemphigus was missing, the collaboration with Enno Schmidt, which is still continuing today, under the supervision of Detlef Zillikens was pivotal to complete our first studies and to get introduced to the pemphigus research community. JW remembers when Detlef was talking with him and his doctoral student Volker Spindler at the International Dermatology Meeting in Los Angeles in 2007, where we were presenting our first poster on pemphigus and he gave us the impression to talk among equal experts. At this occasion, he introduced us to the pioneers of pemphigus research. Afterwards, many discussions and also private talks followed at pemphigus meetings over the years, where Detlef Zillikens advised JW about the strengths and weaknesses of the manuscripts we published with him. For us, Detlef Zillikens was much more than a collaborator and without him and Enno Schmidt, we would not have completed a single study on pemphigus.
