# Supplementary material for: Dsg3 epitope-specific signalling in pemphigus
Source: Front Immunol. 2023 Apr 18;14:1163066. doi: 10.3389/fimmu.2023.1163066 (PMC10151755; doi:10.3389/fimmu.2023.1163066)
Supplement: Supplementary file 1 [file DataSheet_1.pdf]

Supplementary material for: Dsg3 epitope-specific signaling in pemphigus

Thomas Schmitt[1], Christoph Hudemann[2], Sina Moztarzadeh[1], Michael Hertl[2], Ritva Tikkanen[3], Jens Waschke[1]

1: Chair of Vegetative Anatomy, Instiute of Anatomy, Faculty of Medicine, LMU Munich, Germany
2: Department of Dermatology and Allergology, Philipps-University Marburg, Marburg, Germany
3: Institute of Biochemistry, Medical Faculty, Justus-Liebig-University Giessen, Giessen, Germany

First Author: Thomas Schmitt

ORCID: 0000-0003-3781-7236

Corresponding Author: Jens Waschke
ORCID: 0000-0003-1182-5422
Email: jens.waschke@med.uni-muenchen.de
Phone: +4989218072610

Results

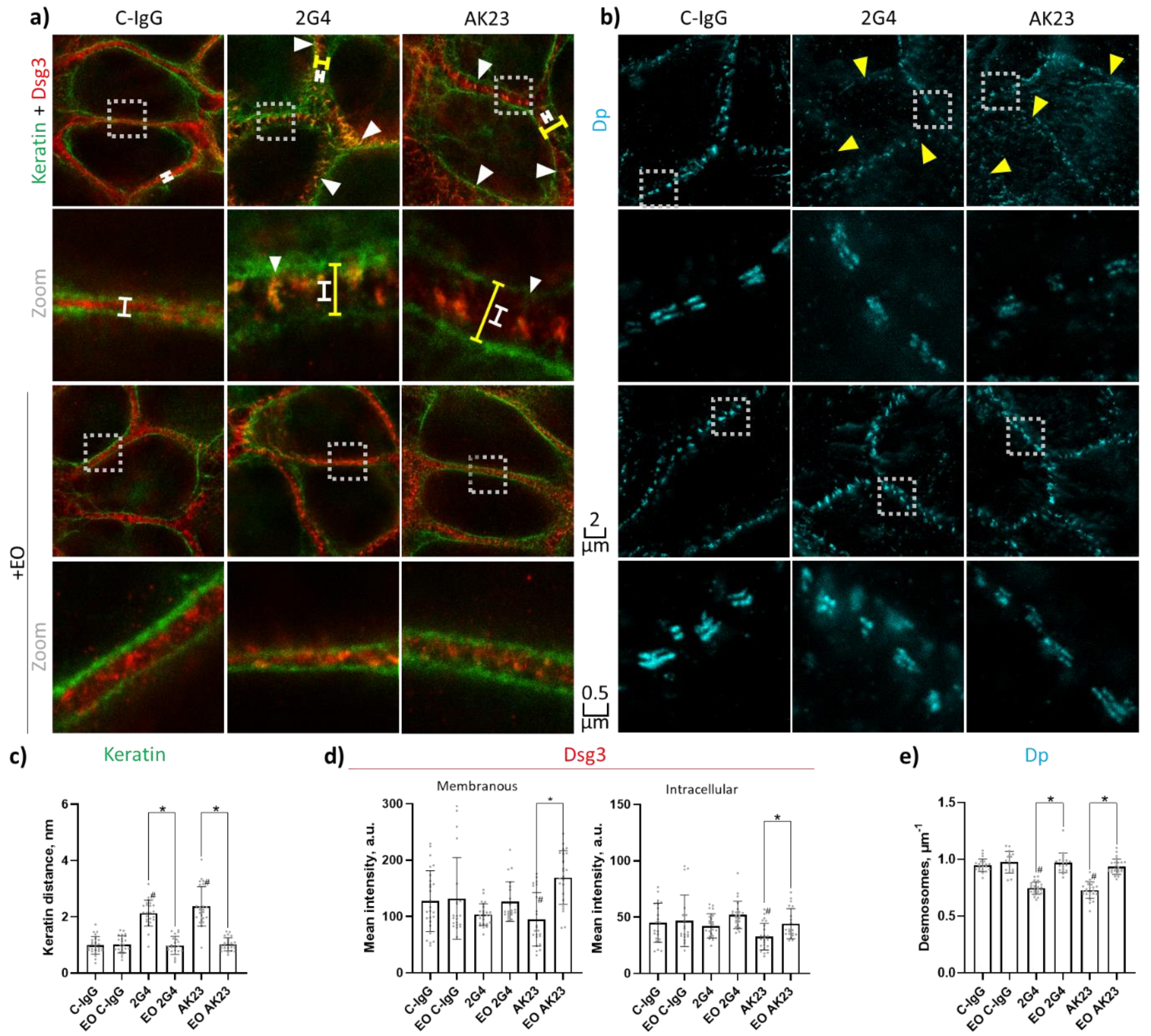

Figure S1: STED microscopy images reveal specific pathogenic effects of 2G4 and AK23. a) Co-staining of Dsg3 (red) and pan-cytokeratin (green) in HaCaT cells treated with either C-IgG, AK23 or 2G4 for 24 h after 1 h preincubation with vehicle or EO (40 nM, inhibiting p38MAPK). Spans illustrate the degree of keratin filament retraction (yellow) compared to control (white). White arrowheads showcase the fragmented staining of Dsg3, which is mostly associated with keratin filaments. b) Staining of Dp (with anti-Dp-pAb, A13299) after 24 h treatment with IgGs and 1 h preincubation with vehicle or EO. Yellow arrowheads indicate reduced numbers of desmosomes per µm of cell border. Two different magnifications are shown for each condition (Scalebars as indicated, boxes with dotted outline indicate the area which was zoomed in on); Quantification of the STED images: c) Keratin filament retraction form the cell borders towards the nucleus. d) Dsg3 staining intensity at the cell borders/membranes (left) or in the cytoplasm (right). e) Number of desmosomes per µm membrane (Experiments (N)=3-4, Evaluated images (n)=12-30). * indicates statistically significant differences between the two conditions indicated, # indicates statistically significant differences towards control conditions, both in two-way-ANOVA with Sidaks correction for multiple comparisons, $p<0.05$.

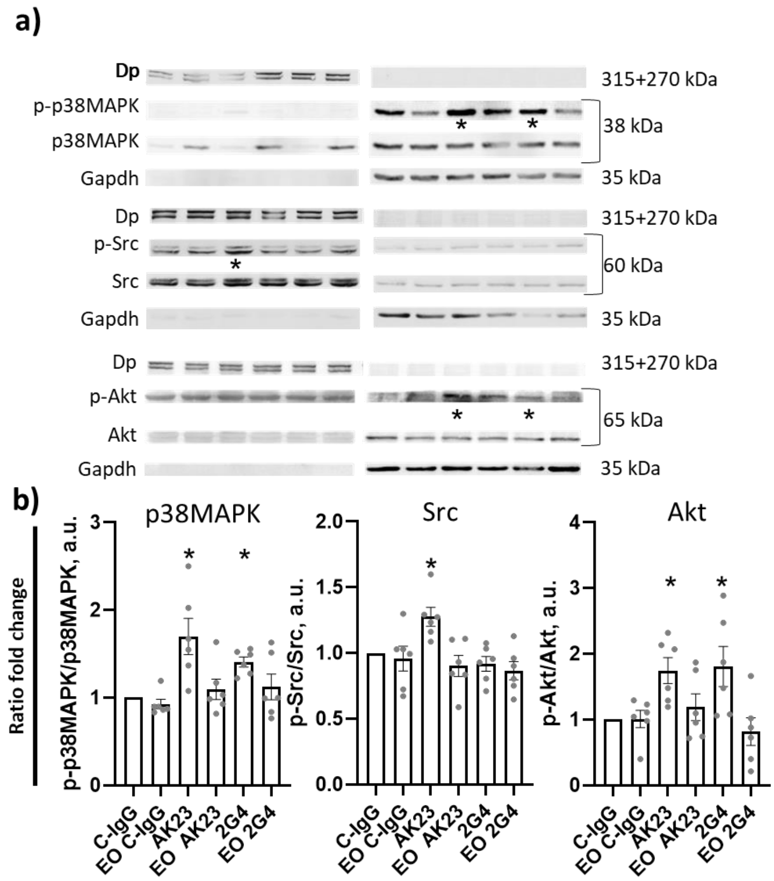

Figure S2: Western blot analysis reveals anti-Dsg3 epitope-specific kinase phosphorylation. a) Representative Western blot images, with respective controls for Triton-soluble (membranous+cytosolic fraction, right) Gapdh and Triton-insoluble (cytoskeleton/desmosome-bound fraction, left) Dp. The cells were treated with either C-IgG, AK23 or 2G4 with or without pre-treatment with EO for 1 h, to inhibit p38 MAPK. b) Quantification of differences in phosphorylation of the investigated proteins from the WB results. Significant changes were observed and quantified in the soluble fraction for p38MAPK and Akt and the insoluble fraction for Src (N=4-6). * marks statistically significant differences between the two conditions indicated in two-way-ANOVA with Sidaks correction for multiple comparisons, p<0.05.

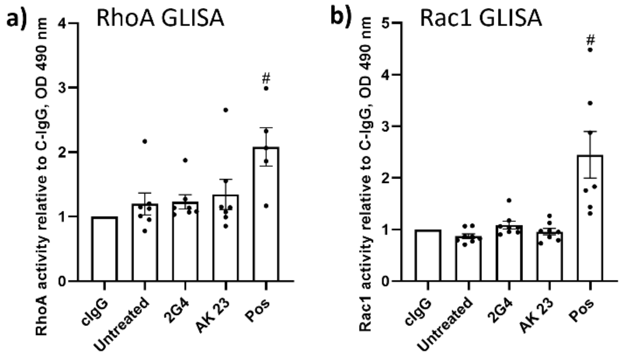

Figure S3: Impact of Dsg3-specific IgGs on RhoA activity. Colorimetric Rho-GTPase ELISA (GLISA) measurement results from HaCaT lysates treated with AK23 2G4 or C-IgG for 2 h. a) For RhoA (N=5-7), b) for Rac1(N=7-8). Pos: positive control = active GTPase provided in the Kit. (# indicate conditions with significant difference, p<0.05, compared to control = c-IgG treated).

a)

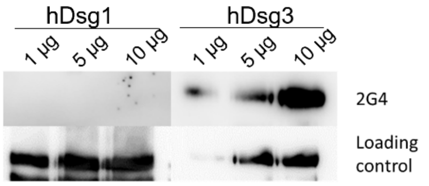

b)

Inhouse	Dsg1					
b(2G4), µg/ml	100	50	10	5	1	0
Abs 405 nm, A.U.	0.119	0.1185	0.1335	0.123	0.1175	0.125

Inhouse	Dsg3					
b(2G4), µg/ml	100	50	10	5	1	0
Abs 405 nm, A.U.	0.9945	1.047	1.0785	1.1355	1.1475	0.121

d)

Euroimmun	Dsg1					
b(2G4), µg/ml	2	0.5	0.2	0.1	0	Pos
Abs 405 nm, A.U.	0.08	0.098	0.097	0.08	0.083	0.235

Euroimmun	Dsg3					
b(2G4), µg/ml	2	0.5	0.2	0.1	0	Pos
Abs 405 nm, A.U.	1.251	1.264	1.129	0.902	0.067	0.207

c)

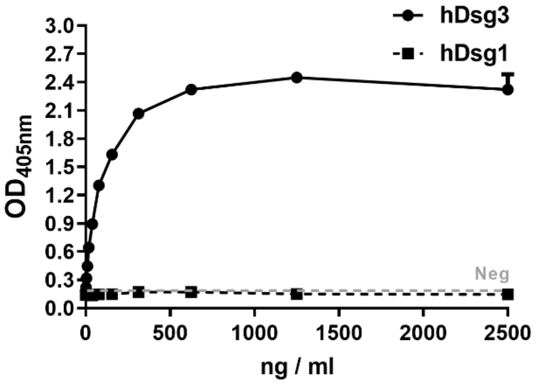

e)

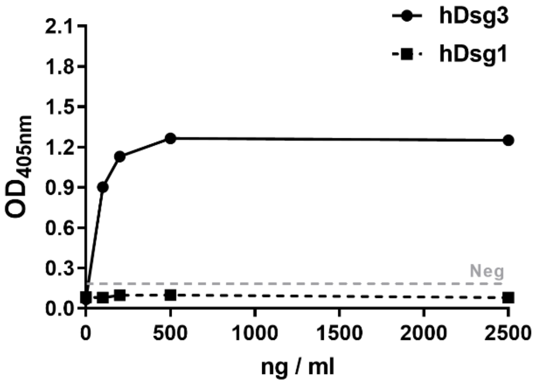

Figure S4: 2G4 Dsg3-specific reactivity. a) Western-Blot results with 2G4 recognizing hDsg3 but not hDsg1. Loading control: anti-E-Tag for hDsg1 and anti-His-Tag for hDsg3b, c) inhouse ELISA results for binding of Dsg3 and Dsg1 by 2G4. d, e) modified commercially available anti-Dsg1 and anti-Dsg3 ELISA (Euroimmun).

Materials and Methods

Protein analysis by Western blot

Full length human Dsg1 and 3 (extracellular domain) carrying an E-Tag (hDsg1) or a His-Tag (hDsg3) were produced in baculovirus infected insect cells (High Five; Invitrogen, CA, USA) as described previously[1]. Recombinant proteins were purified by affinity chromatography using nickel-nitrilotriacetic agarose beads (Qiagen, DE) according to manufacturer's instructions. Specific detection was performed by Western blot analysis using the primary murine antibody 2G4 (10 µg/ml) and indicated protein concentrations. Horseradish peroxidase (HRP)-conjugated anti-mouse IgG (1:1000; Dako, Denmark) served as secondary antibody. For loading control, a monoclonal rabbit anti-E-Tag antibody (1:6000; Abcam, UK) (for hDsg1) or rabbit anti-His-Tag antibody (1:2000; Daco) (for hDsg3) were used. Antibody binding was visualized by using a commercial HRP substrate (Immobilon Western Chemiluminescent HRP substrate; Millipore, USA). Signals were detected with a digital chemiluminescence reader (PEQLAB, DE).

ELISA

For antigen-dependent antibody quantification, 10 µg/ml recombinant hDsg3 or hDsg1 diluted in PBS was coated onto immunomicrotitre plates (96-Well; Greiner Bio-One, DE) (inhouse protocol). 2G4 served as primary antibody at indicated concentrations. For visualization, HRP-conjugated antibodies (1:2000, Dako, DNK) were used. Absorbance level was measured at 405 nm (Tecan plate reader Sunrise + Magellan software; Tecan Group Ltd., Männedorf, CH). Additionally, autoantibody titers were analysed at indicated concentrations using both the commercially available anti-Dsg1 and anti-Dsg3-ELISA (Euroimmun, DE)

Colorimetric G-LISA Rac1 and RhoA activity measurements

The intracellular GTP-bound Rac1 GTPase and RhoA GTPase concentration was measured by using a G-protein ELISA (Cytoskeleton, USA.). HaCaT cells were seeded in 6 well plates and grown to confluency. They were treated with IgGs (AK23, 2G4 or C-IgG) for 2 h. Afterwards they were processed according to the manufacturer's instructions. After the reaction, the absorbance at a wavelength of 490 nm was measured using a TECAN, Infinite 200 PRO microplate reader (Tecan GmbH, DE).

Table S1: Antibodies used

Antibody	Catalog No.	Company	Concentrations
anti-Dsg3-pAb	62720-120	Biozol, DE	1:200 IF
anti-cytokeratin-pan-mAb	C2931	Sigma Aldich, USA	1:200 IF
anti-Dp-pAb	A7169	Abcam, Britan	1:200 IF
anti-Dp-pAb	A13299	Abcam, Britan	1:200 IF
p-p38MAPK	4511	Cell signaling, USA	1:500 WB
p38MAPK	9212	Cell signaling, USA	1:500 WB
p-Src	2105	Cell signaling, USA	1:1000 WB
Src	2109	Cell signaling, USA	1:1000 WB
p-Akt	4060	Cell signaling, USA	1:1000 WB
Akt	9272	Cell signaling, USA	1:1000 WB
anti-Gapdh-mAb	sc-47724	Santa Cruz, USA	1:4000 WB

Table S2: Signalling pathway inhibitors

Chemical	Target	solvent	Final concentration	Company
BTP-2	CRAC inhibitor	PBS/DMSO 1:20	10 µM	Merk, DE
GSK-F1	PI4K inhibitor	PBS/DMSO 1:20	10 nM	SYNkinase, AU
U-73122	PLC inhibitor	PBS/DMSO 1:2	4 µM	Santa Cruz, USA
Xestospongin C (Xest)	IP3R inhibitor	DMSO	2 µM	Abcam, USA
SB202190	p38MAPK inhibitor	PBS/DMSO 1:10.000	60 µM	Sigma Aldich, USA
EO1428	p38MAPK inhibitor	PBS/DMSO 1:10.000	40 nM	Torcis, UK
PP2	Src inhibitor	PBS/DMSO 1:10.000	10 µM	Calbiochem, GDE

References

1. Müller R, Svoboda V, Wenzel E, Müller H-H, Hertl M. IgG against extracellular subdomains of desmoglein 3 relates to clinical phenotype of pemphigus vulgaris. *Exp Dermatol*. 2008;17(1):35-43. doi:10.1111/j.1600-0625.2007.00615.x.
